# Evaluation of peripheral binocular visual field in patients with glaucoma: a pilot study

**Published:** 2016

**Authors:** Banc Ana, Stan Cristina, Chiselita Dorin

**Affiliations:** * Ophthalmology Clinic, County Clinical Emergency Hospital of Cluj-Napoca, Cluj, Romania; “Iuliu Hatieganu” University of Medicine and Pharmacy Cluj-Napoca, Cluj, Romania; ** Ophthalmology Clinic, “Sf. Spiridon” Emergency Hospital Iasi, Romania; “Grigore T. Popa” University of Medicine and Pharmacy Iasi, Romania

**Keywords:** Peripheral visual field, Binocular visual field, Glaucoma, Threshold strategy

## Abstract

Objective: The objective of this study was to evaluate the peripheral binocular visual field (PBVF) in patients with glaucoma using the threshold strategy of Humphrey Field Analyzer. Methods: We conducted a case-control pilot study in which we enrolled 59 patients with glaucoma and 20 controls. All participants were evaluated using a custom PBVF test and central 24° monocular visual field tests for each eye using the threshold strategy. The central binocular visual field (CBVF) was predicted from the monocular tests using the most sensitive point at each field location. The glaucoma patients were grouped according to Hodapp classification and age. The PBVF was compared to controls and the relationship between the PBVF and CBVF was tested.

Results: The areas of frame-induced artefacts were determined (over 50° in each temporal field, 24° superiorly and 45° inferiorly) and excluded from interpretation. The patients presented a statistically significant generalized decrease of the peripheral retinal sensitivity compared to controls for Hodapp initial stage - groups aged 50-59 (t = 11.93 > 2.06; p < 0.05) and 60-69 (t = 7.55 > 2.06; p < 0.05). For the initial Hodapp stage there was no significant relationship between PBVF and CBVF (r = 0.39). For the moderate and advanced Hodapp stages, the interpretation of data was done separately for each patient. Conclusions: This pilot study suggests that glaucoma patients present a decrease of PBVF compared to controls and CBVF cannot predict the PBVF in glaucoma.

**Abbreviations:** CBVF = central binocular visual field, PBVF = peripheral binocular visual field, MD = mean deviation

## Introduction

The amount of binocular visual field loss in glaucoma was extensively investigated, considering its influence on the quality of life and the activities of daily living [**1**-**5**]. Both central and peripheral visions were investigated, but the algorithm to evaluate the binocular visual field is still to be determined as there is no common protocol on the strategy of testing or on the extent of the evaluated visual field [**6**-**12**].

The purpose of our study was to evaluate the peripheral binocular visual field (PBVF) in patients with glaucoma using the threshold strategy of Humphrey Field Analyzer. We designed a reproducible custom test and compared its results with controls and with the central binocular visual field (CBVF) test results of the patients themselves.

## Materials and methods

**Participants**

We conducted a case-control pilot study in which we enrolled 59 patients with various degrees of glaucomatous damage and 20 non- glaucomatous patients, who presented in the outpatient department of Ophthalmology of a tertiary care hospital. Informed consent was obtained from all 79 participants prior to testing. Our research adhered to the tenets of the Declaration of Helsinki.

The inclusion criteria for the cases were: confirmed diagnosis of glaucoma (based on the presence of optic nerve head’s cup/ disc ratio above 0.3; intraocular pressure above 21 mmHg measured with Goldmann aplanation tonometry; visual field results of Glaucoma Hemifield Test ‘outside normal limits’ and a minimum of three clustered points with significantly depressed sensitivity, of which one with p<1%); absence of other ocular disease (e.g., corneal opacity, active uveitis, moderate/ dense cataract, vitreous deposits, retinal detachment, age-related macular degeneration, hypertensive retinopathy, diabetic retinopathy, retinal laser treatment, optic neuropathy other than glaucoma, amblyopia); absence of stroke or other known brain injuries (that may influence the results of the visual field testing); at least 3 central visual field tests performed in the past; all of the visual tests with false positive and false negative errors less than 10%; spherical refractive errors less than 6 diopters; cylindrical refractive errors less than 3 diopters. Patients with incipient cataract or intraocular lens were not excluded.

The non-glaucomatous patients were considered controls and were enrolled in our study if they did not present any ocular finding except for incipient cataract, intraocular lens, spherical refractive errors less than 6 diopters or cylindrical errors less than 3 diopters. The optic nerve head appearance was not suggestive for glaucoma, intraocular pressure was below 21 mmHg measured with Goldmann aplanation tonometry in the absence of ocular hypotensive treatment; visual field results of Glaucoma Hemifield Test were ‘within normal limits’.

**Visual field testing**

All visual field tests were performed for both cases and controls using the Humphrey Field Analyzer (HFA II, Carl Zeiss Meditec, Dublin, CA), as follows: one monocular Central 24-2 Threshold Test for each eye, Swedish Interactive Threshold Algorithm - Fast strategy, and one peripheral binocular custom test. We established the reliability criteria for the central monocular tests as: fixation losses ≤ 25%; false positive errors ≤ 10%; false negative errors ≤10%.

The CBVF was obtained from the results of the two monocular central tests using the best location model, which states that for corresponding visual field locations, the binocular sensitivity is given by the most sensitive location between the two eyes [**7**]. Monocular tests were performed using the lens correction indicated by the Field Analyzer based on the patient’s refraction. The lens was placed in front of the tested eye into the lens holder. An eye patch was placed over the non-tested eye. The scores of the retinal sensitivities as given on the printout for each point of the monocular test were manually introduced into a spreadsheet (Microsoft Excel; Microsoft Corporation, Redmond, WA) and combined using an algorithm which selected for each binocular point the highest value of the two corresponding monocular points. The values of the retinal sensitivities were expressed in decibels, as the values are given in decibels by the Field Analyzer.

**Fig. 1:**
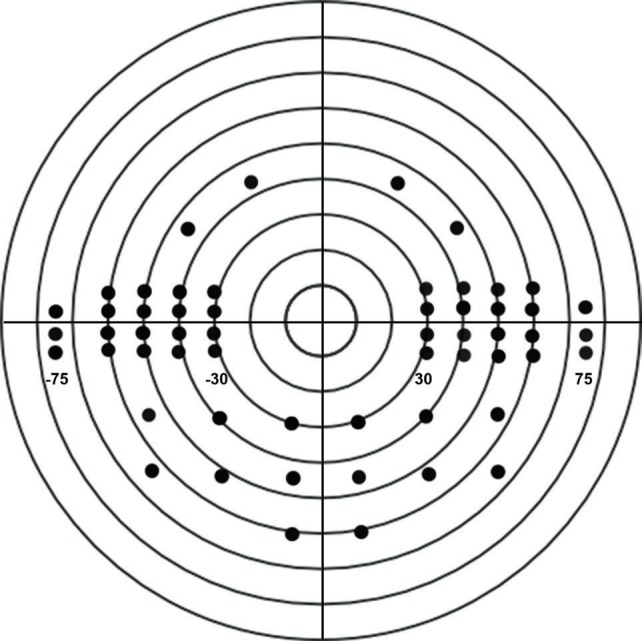
The peripheral binocular custom test pattern

We created the peripheral custom test by selecting System Setup from the Main Menu, then Additional Setup, then Custom Test and Create Threshold Test. Our peripheral test evaluated 54 points that extended from 30° to 75° in each temporal field, from 30° to 60° inferiorly and to 45° superiorly, as seen in **Fig. 1**. These points correspond to the region located at more than 30° from the fixation point of the Esterman test pattern.

Because the custom test was performed using the threshold strategy, appropriate lens correction was needed [**13**]. The perimeter’s lens holder is designed for testing one eye at a time, so a custom-made trial frame was built in order to decrease to a minimum the frame-induced artefacts by using the minimum thickness of the frame components. The test was performed using the lens correction indicated by the Field Analyzer based on the patient’s refraction for each eye; standard trial lenses were used. The Fixation Monitoring selection was ‘Off’ and the video eye monitor was aligned to the bridge of the nose. The participants were monitored throughout the test and were instructed to maintain the central fixation with both eyes. The reliability criteria for the peripheral test were: false positive errors ≤ 25%; false negative errors ≤ 25%. The printout contained only the numeric values (expressed in decibels) for each tested point, without gray scale, defect depth or other analysis available for a standard visual field test.

**Statistical analysis**

Based on the central monocular visual fields, the results were sorted according to Hodapp classification [**14**] and then by age. Briefly, the Hodapp classification stages the visual field loss as early, moderate and advanced based on the mean deviation (MD) and the number and location of points with different values of depressed retinal sensitivity.

For each age group and Hodapp stage, the PBVF was compared to controls using two-tailed paired t test and Pearson correlation coefficient. The output of statistical analysis was expressed as t-value and Pearson coefficient, considering a p-value less than 0.05.

For each Hodapp stage the relationship between the PBVF and CBVF was tested using the correlation coefficient (r). The parameter used for PBVF was the sum of the peripheral retinal sensitivities expressed in decibels. The parameters used for CBVF were the maximum MD between the eyes and the sum of the central retinal sensitivities expressed in decibels.

## Results

The baseline characteristics of the participants are summarized in **Table 1**.

**Table 1:** Baseline characteristics of the participants

	Cases	Controls
Age (years)		
Mean ± standard deviation	64.1 ± 7.97	60.7 ± 11.5
Range	43 to 79	40 to 78
Sex		
Male	16 (27%)	4 (20%)
Female	43 (73%)	16 (80%)
Diabetes	4 (7%)	0
Hypertension	15 (25%)	3 (15%)
Thyroid disease	2 (3%)	0
Arthritis	8 (13%)	2 (10%)

**Fig. 2:**
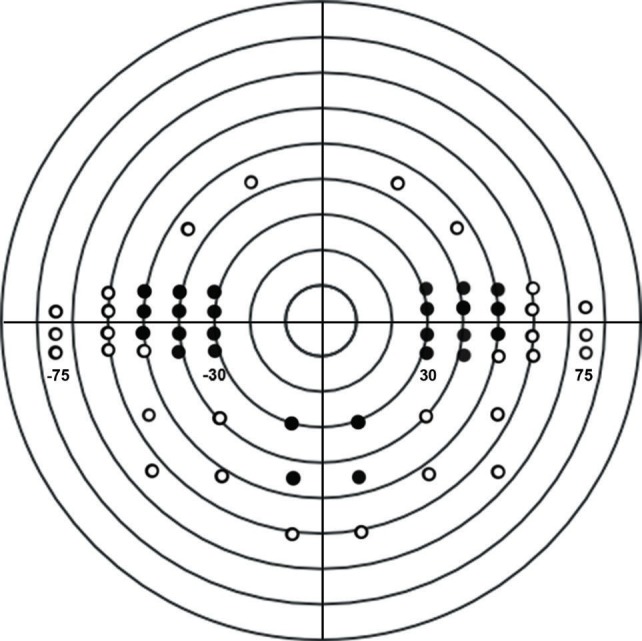
The empty circles represent the peripheral areas within points with no retinal sensitivity were found

Examining the PBVF test results, we noticed areas with no retinal sensitivity for both cases and controls: there were at least 3 points without ret- inal sensitivity for 88% of the cases (52 cases out of 59) and for 80% of controls (16 controls out of 20). These points were located in the regions represented in **Fig. 2**. As they were surrounded by points with retinal sensitivity, we considered these peripheral deficits as frame-induced arte- facts and decided to eliminate the entire region from statistical interpretation. The resultant bin- ocular visual field extends to 50° in each temporal field, 24° superiorly and 45° inferiorly.

The distribution of patients according to Hodapp stage and decade of age is presented in **Table 2**.

**Table 2:** Distribution of participants according to Hodapp classification and age

Decade	Hodapp initial stage	Hodapp moder- ate stage	Hodapp advanced stage	Con- trols
40 – 49 years	1 patient			5 partic- ipants
50 – 59 years	14 pa- tients	1 patient		5 partic- ipants
60 – 69 years	23 pa- tients	1 patient	2 patients	5 partic- ipants
70 – 79 years	14 pa- tients	1 patient	2 patients	5 partic- ipants

The results of the tested correlation between the sums of peripheral retinal sensitivities obtained for the patients and for the controls from the corresponding decades are comprised in **Table 3**.

**Table 3:** Correlation between the PBVF parameters of Hodapp initial stage glaucoma patients and controls

	t-value (two-tailed paired t test)	p-value	Pearson coefficient
50 - 59 years	11.93 (>2.06)	<0.0001	0.91
60 - 69 years	7.55 (>2.06)	<0.0001	0.80
70 - 79 years	1.09 (<2.06)	0.28	0.88

There is a statistical significant difference for the groups of 50-59 and 60-69 years of age, meaning that the peripheral retinal sensitivity is lower in patients with glaucoma compared to normal participants. For the same age groups, the Pearson coefficient is high, meaning a high correlation between the variation of peripheral retinal sensitivities in patients and normal participants, or in other words, the decrease of the peripheral retinal sensitivity in patients with glaucoma is generalized.

Because of the size of the samples, the results derived from the other age groups and Hodapp stages were analyzed separately, by comparison with the results of the controls. For all 8 cases, we observed a general decrease of the peripheral retinal sensitivity.

For the decades 50-59, 60-69 and 70-79 included in the initial Hodapp stage, the correlation coefficient between the maximum MD and the sum of peripheral retinal sensitivities was r = 0.32 and the correlation coefficient between the sum of the central and peripheral retinal sensitivities was r = 0.39, indicating the absence of correlation between the parameters of CBVF and PBVF.

## Discussion

The visual field loss in glaucoma was markedly investigated, given its impact on the quality of life [**1**-**5**]. Owen et al. [**2**] focused their research on the binocular visual field as a measure of predicting the visual loss to a level below the legal standard for driving. Kulkarni et al. [**5**] compared eight methods of staging visual field damage in glaucoma with a performance- based measure of the activities of daily living and self-reported quality of life; their conclusion was that the most accurately predictors for functional ability and quality of life in glaucoma were the amount of binocular visual field loss and the status of the better eye.

Regarding the central visual field, Crabb and Viswanathan [**9**] described a method of merging the results from monocular fields to obtain the integrated visual field. Nelson-Quigg et al. [**7**] compared four models of prediction of CBVF from the monocular results and concluded that the binocular summation and best location models provided the best predictions. All of these tests were performed using the threshold strategy of the Humphrey Field Analyzer; the latter corresponds to the method described by Crabb and Viswanathan [**9**] and is the method we used in our study to obtain the CBVF.

The peripheral binocular visual field was investigated by two methods of computerized perimetry: the Esterman binocular test [**8**-**10**,**15**] and peripheral custom tests [**8**,**15**]. The Esterman binocular test is the only standard binocular test available on Humphrey Field Analyzer [**16**] and it is using a non-adjustable high level of stimulus brightness which is unable to detect subtle defects of the visual field. A 10 decibels stimulus is presented in 120 points of the visual field to an extent of 150° bilateral horizontal field width, with more points being tested in the inferior field than superiorly [**16**]. The results of the Esterman binocular test were compared to the results of custom tests which also used a non-threshold strategy: Jampel et al. [**8**] designed two custom peripheral binocular visual fields using non-adjustable levels of brightness of the stimuli, but with a decreased intensity compared to the intensity used for the Esterman test - 20 and 22 decibels, respectively. Their results indicate that the custom tests provide a wider range of responses compared to Esterman binocular test, but do not correlate better with patient assessment of vision, suggesting the need of a better method of testing, such as threshold strategy [**8**]. The objective of threshold testing is to determine the differential sensitivity for each retinal point tested; the stimuli are either dimmed or made brighter in steps until the patient marks the seen stimulus [**16**].

Morescalchi et al. [**15**] designed another custom binocular program in order to quantify peripheral visual impairment. It was used the screening 3-zone strategy, which provides only certain symbols for the seen stimuli, relative defects and absolute defects, without the retinal sensitivity values [**13**]. However, the authors mention that the new test was proposed for evaluation of visual impairment only for legal purposes; its results correlated better with patient-reported assessment of vision in comparison with binocular Esterman test [**15**].

According to European Glaucoma Society’s guidelines [**17**], threshold strategy of computerized perimetry is the recommended standard for evaluation of glaucoma patients. The central 30° visual field is most investigated because this central area corresponds to the location of the great majority of retinal ganglion cells [**17**]. The PBVF is evaluated in most cases for legal purposes. However, the peripheral visual tests available on computerized perimetry can detect only the advanced defects [**8**,**10**,**15**,**16**] and most of the information about the peripheral visual field in glaucoma was obtained using kinetic perimetry [**17**].

We designed this pilot study to test the PBVF of patients with glaucoma with a reproducible custom binocular test using the threshold strategy of Humphrey Field Analyzer. We did not perform peripheral monocular visual field tests in order to integrate them into one PBVF as to the best of our knowledge, there is no such model for the peripheral visual field tested with computerized perimetry.

The results of our study suggest that glaucoma patients present a decrease of PBVF compared to controls for the patients aged 50-59 and 60-69 included in Hodapp initial stage. Moreover, the pattern of this decrease is generalized. This result indicates the need of peripheral visual field evaluation in patients with glaucoma, not only for the advanced cases, but also for the Hodapp initial stage, according to the classification based on the results of the central monocular tests.

The fact that we did not find a correlation between the parameters of PBVF and CBVF suggests that the status of the central visual field cannot predict the status of the peripheral visual field, indicating again the need of peripheral visual field evaluation in patients with glaucoma.

The presence of frame-induced artefacts we observed in our study raises the question of the functional impact of the eyeglasses in every day life. From the patient’s point of view, wearing eyeglasses may be a part of functional binocular visual field, but from the researcher’s point of view, it is difficult to assess the role of the frames on the visual field as the manufacture of the trial frame is restricted by the size and shape of trial lenses available. Nelson-Quigg et al. [**7**] used in their study a modified pediatric trial frame for binocular testing, but their test examined only the central 30° visual field and no frame-induced artefacts were reported [**7**]. According to the Humphrey Field Analyzer User Manual [**13**], it is needed to use the patient’s glasses to perform the Esterman binocular test if the patient does require glasses for the activities of daily living. Among the studies we found that used the Esterman binocular test [**5**,**8**,**10**] no information is provided about optical correction or the potential frame- induced artefacts for this test.

One limitation of our study may be due to the non-standardized trial frame we used for the evaluation of the PBVF, although its manufacture was being influenced by the diameter of the standard trial lenses.

The small number of patients included in our study in moderate and advanced Hodapp stages is a consequence of the restrictive inclusion criteria, as these patients usually have other ocular findings that can influence the test results. We established restrictive inclusion criteria because we decided to investigate the effects of glaucoma alone on binocular visual field.

The difficulty in enrolling the participants was even greater with the controls than with the cases. We found challenging to exclude glaucoma and other ocular or brain conditions which may alter the visual field test results in a person aged more than 50. Moreover, a glaucoma patient has learnt during the numerous follow-up visits how a visual field test is performed and the importance of this examination, whereas a non-glaucomatous patient may not have the motivation to complete the test.

In conclusion, our pilot study suggests that glaucoma patients present a generally depressed PBVF compared to controls and CBVF cannot predict the PBVF in glaucoma. These results indicate the requirement of peripheral visual field assessment in glaucoma using the threshold strategy.

**Financial disclosure None**

## References

[1] Medeiros FA, Weinreb RN, Boer ER, Rosen PN (2012). Driving Simulation as a Performance based Test of Visual Im- pairment in Glaucoma. J Glaucoma.

[2] Owen VMF, Crabb DP, White ET, Viswanathan AC, Gar-way-Heath DF, Hitchings RA (2008). Glaucoma and Fitness to Drive: Using Binocular Visual Fields to Predict a Mile- stone to Blindness. Invest Ophthalmol Vis Sci.

[3] Crabb DP, Fitzke FW, Hitchings RA, Viswanathan AC (2004). A practical approach to measuring the visual field compo- nent of fitness to drive. Br J Ophthalmol.

[4] McGwin G, Huisingh C, Jain SG, Girkin C, Owsley C (2015). Bin- ocular Visual Field Impairment in Glaucoma and At-fault Motor Vehicle Collisions. J Glaucoma.

[5] Kulkarni KM, Mayer JR, Lorenzana LL, Myers JS, Spa-eth GL (2012). Visual Field Staging Systems in Glaucoma and the Activities of Daily Living. Am J Ophthalmol.

[6] Asaoka R, Crabb DP, Yamashita T, Russell RA, Wang YX, Garway-Heath DF (2011). Patients Have Two Eyes!: Binocular versus Better Eye Visual Field Indices. Invest Ophthal- mol Vis Sci.

[7] Nelson-Quigg JM, Cello K, Johnson CA (2000). Predicting Binoc- ular Visual Field Sensitivity from Monocular Visual Field Results. Invest Ophthalmol Vis Sci.

[8] Jampel HD, Friedman DS, Quigley H, Miller R (2002). Correlation of the Binocular Visual Field with Pacient Assessment of Vision. Invest Ophthalmol Vis Sci.

[9] Crabb DP, Viswanathan AC (2005). Integrated visual fields: a new approach to measuring the binocular field of view and visual disability. Graefe’s Arch Clin Exp Ophthalmol.

[10] Crabb DP, Viswanathan AC, McNaught AI, Poinoosaw- my D, Fitzke FW, Hitchings RA (1998). Simulating binocular visual field status in glaucoma. Br J Ophthalmol.

[11] Crabb DP, Smith ND, Glen FC, Burton R, Garway-Heath DF (2013). How Does Glaucoma Look?. Patient Perception of Vi- sual Field Loss. Ophthalmology.

[12] Gestel A, Webers CAB, Beckers HJM, Dongen MCJM, Sev- erens JL, Hendrikse F, Schouten JSAG (2010). The relationship between visual field loss in glaucoma and health-related quality-of-life. Eye.

[13] Carl Zeiss Meditec Inc (2003). Humphrey Field Analyzer II-I se-ries, User’s Guide.

[14] Hodapp E, Parrish RK, Andersson DR (1993). Clinical decisions in glaucoma.

[15] Morescalchi F, Gandolfo E, Gandolfo F, Zinzini E (2003). Periph-eral low vision quantification: comparison between two binocular programs in Humphrey perimetry. Minerva Oftalmol.

[16] Heijl A, Patella VM, Bengtsson B (2012). Effective Perimetry. The Field Analyzer Primer.

[17] European Glaucoma Society (2014). Terminology and guide- lines for glaucoma.

